# Determination of the patient safety culture among nurses working at intensive care units

**DOI:** 10.12669/pjms.313.7059

**Published:** 2015

**Authors:** Zuhal Yilmaz, Songul Goris

**Affiliations:** 1 Zuhal Yilmaz, Yahyali Health Vocational School, Kayseri-Turkey; 2 Songul Goris, RN, PhD. Assistant Professor, Department of Internal Diseases Nursing, Erciyes University, Faculty of Health Sciences, 38039, Kayseri-Turkey

**Keywords:** Patient safety, Intensive Care Unit, Nursing

## Abstract

**Objective:**

To determine the patient safety culture among nurses working at intensive care units.

**Methods:**

This descriptive study was conducted at intensive care units of Health Practice and Research Center of Erciyes University and Kayseri Education and Research Hospital in the city center of Kayseri in Turkey. Three hundred sixteen nurses working at intensive care units at these hospitals were included in the study. Data were collected by using Hospital Survey on Patient Safety Culture (HSOPSC), developed by Agency for Healthcare Research and Quality. Percentage distribution and Mann Whitney U Test were used to analyze the data.

**Results:**

About 13.6% of the nurses working at intensive care units stated that they faced incidents of potential threat to the patient safety and that 48.8% of these cases were falls. Although a great majority of the nurses (88%) indicated that they never documented a case report, they assessed the patient safety in their institution as acceptable (43%). Out of the 12 dimensions of Hospital Survey on Patient Safety Culture, the percentage of positive responses was the highest for “teamwork within units” dimension and lowest for the “non-punitive response to error” dimension.

**Conclusion:**

Awareness of the nurses regarding patient safety should be raised and their related knowledge should be kept up-to-date through more frequent in-service trainings.

## INTRODUCTION

Prevention of healthcare-related errors and reduction or elimination of patient problems caused by such errors is based on formation of the patient safety culture. Therefore, patient safety culture is important in terms of representation of quality healthcare services.^1^,^2^ The patient safety involves all measures and precautions made for reduction or elimination of possible adverse effects of medical care during medical diagnosis and treatment.

According to a report released by the Institute of Medicine (IOM), 44.000 to 98.000 people die every year as a result of a medical error. Number of deaths caused by medical errors is ranked as the eighth, which is followed by Acquired Immune Deficiency Syndrome (AIDS), breast cancer and traffic accidents.^3^ The most frequent problems threatening the patient safety are diagnosis errors, medication errors, hospital infections, bedsores, complications during and after the operation, errors induced by breakdown of equipments-appliances, falls and ventilator-related errors.^4^,^5^

The facilities and properties of the hospital units can also pose serious risks in terms of medical errors which can be threatening to the patient safety. Intensive care units (ICUs) are privileged departments where more serious patients requiring constant monitoring are provided care and many advanced technological life-saving equipment and appliances are used.^6^ It is possible for all patients admitted to ICUs to face a life-threatening error during their hospitalization.^1^

Nurses working at intensive care units have a crucial role in the establishment of a safe and qualified care for the patients. ICU nurses are the personnel who give constant care, apply complicated medications, use various technological equipments, and offer care to patients in need of advanced life support.^7^,^8^ For this reason, it is of great significance for the nurses to adopt, defend, and have a critical perspective on the issue of patient safety to offer a prolonged and safe care.^7^,^9^

It is required to determine primarily the patient safety culture in the institution in order to enhance the patient safety culture and prevent deficiencies, practices or risk factors causing medical errors.^10^,^11^ The purpose of this study was to assess perspectives of nurses regarding patient safety culture and their related knowledge and practices.

## METHODS

This descriptive study was conducted at intensive care units of Health Practice and Research Center of Erciyes University and Kayseri Education and Research Hospital in the city center of Kayseri in Turkey. The population of the study consisted of 399 nurses working at intensive care units at these hospitals. 316 of these nurses constituted the sample group of this study. No systematic sampling was done and all the voluntary nurses on duty between November 2012 and December 2012 were included in the study. Data were collected by using a questionnaire and Hospital Survey on Patient Safety Culture (HSOPSC)^12^, developed by The Agency for Healthcare Research and Quality (AHRQ). Developed by the AHRQ^12^ with the aim of determining the patient safety culture in hospitals in USA, HSOPSC was adapted to the Turkish by Bodur and Filiz,^13^ and its validity and reliability study was conducted. The questionnaire, designed by the researchers upon a comprehensive survey of literature, involved socio-demographic characteristics of the nurses and included questions about the patient safety.^1^,^9^,^13^

The HSOPSC has 42 items measuring 12 dimensions of the safety culture. It contains 18 negatively-worded items on safety culture and 7 items on personal information. It measures the dimensions of patient safety culture in hospitals in general as well as in hospital units in particular. For each positively worded item, the percentage of positive responses is calculated by using the percentage of respondents answering the question as “strongly agree”, “agree”, “always” or “most of the time”. Likewise, for each negatively worded item, the percentage of negative responses is calculated by making reverse coding. The obtained scores indicate the percentage of positive responses. Dimension scores are calculated by taking the mean scores of the dimension items. In all cases, the possible range of score is from 0 to 100, with higher scores indicating more positive safety culture.^12^,^13^ The Cronbach’s alpha reliability coefficient was determined as 0.79 for this study.

Ethics committee approval was received from Erciyes University Clinical Research Ethics Board and official permissions were obtained from the institutions where the study was conducted. In addition, the participating nurses submitted their written and verbal informed consents. In this study, the normal distribution of the data was analyzed through Shapiro-Wilk test. While percentage distribution was used to assess the data, Mann-Whitney U was used to compare difference between two groups. The summary statistics are shown as number of unit (*n*), percentage (%) and *median (25p- 75p)*. Statistical significance was set at *p*<0.05.

## RESULTS

The average age of the nurses was 30.4±5.3, 80.4% of them were female, and 65.8% were married. 67.4% of the nurses held a Bachelor degree, and 44.6% had a nursing experience of 2 - 5 years. 25.6% stated that they worked at neonatal intensive care units. About half of the nurses (50.3%) reported that they were satisfied with their jobs. Table-I illustrates knowledge and practices of the nurses about the patient safety. It was observed that a majority of the nurses (69.6%) got training on patient safety, and that 13% encountered an incident of threat to patient safety. In addition, 48.8% assessed falls as the most common case they encountered, and nearly all of the nurses (93%) indicated that they shared incidents of patient safety with the medical team.

**Table-I:** Nurses’ patient safety knowledge and practices (n=316).

Knowledge Level and Practices	n	%
*Patient safety training*		
Received	220	69.6%
Not received	96	30.4%
*Duration of receiving patient safety training*		
1 year and under	105	47.7%
2 to 4 years	88	40.0%
5 years and over	27	12.3%
*Knowledge about patient safety rules and regulations*		
Knowledgeable	112	35.4%
Not knowledgeable	204	64.6%
*State of encountering incidents of potential threat to patient safety*		
Yes	43	13.6%
No	273	86.4%
*Incidents of potential threat to patient safety*		
Falls	21	48.8%
Medications	7	16.3%
Infections	7	16.3%
Lack of equipment	5	11.6%
Negligence of treatment or communication-related errors	3	7.0%
*The time of the safety-threatening incidents*		
In the last year	27	62.8%
In the last two years or before	16	37.2%
Responses to the safety-threatening incidents *		
Sharing with the medical team	40	93.0%
Taking measures following the incident	26	60.5%
Giving information to the patient and his/her relatives	14	32.5%
Filling out a case report	7	16.3%
Keeping the incident as secret	2	4.7%
*Reasons for the threats to patient safety (n=242)*		
Heavy workload	135	55.8%
Working in stressful environment	54	22.3%
Dealing with patients in need of complicated care	53	21.9%

* More than one response is given.

Table-II shows the percentages of positive responses for dimensions of HSOPSC. As indicated in the table, “teamwork within units” among the 12 dimensions had the highest percentage (80.3%) whereas “nonpunitive response to error” had the lowest percentage (25.1%).

**Table-II:** Means of positive responses on patient safety culture dimensions (n=316).

Dimensions	Means of positive responses (%)
Overall perceptions of patient safety	64.9%
Teamwork across hospital units	49.4%
Hospital handoffs and transitions	59.4%
Manager expectations and actions promoting safety	40.8%
Organizational learning and continuous improvement	59.4%
Teamwork within units	80.3%
Communication openness	44.9%
Feedback and communication about error	55.1%
Non-punitive response to error	25.1%
Staffing	33.8%
Management support for patient safety	38.7%
Frequency of events reported	25.9%

When ratings of the nurses of patient safety in their units were analyzed, it was seen that 43% assessed their units as “acceptable” in terms of patient safety. About 13.6% of the nurses working at intensive care units stated that they encountered incidents of potential threat to the patient safety and that 48.8% of these cases were falls. The results also indicated that a great majority of the nurses (88%) did not fill out a case report on patient safety in the last 12 months.

Table-III illustrates a comparison of perceptions of the nurses regarding the dimensions of the patient safety culture based on whether they received training or not. The mean scores obtained by trained nurses from the dimensions “handoffs and transitions” (*p*=0.017) and “frequency of events reported” (*p*=0.003) were higher compared to the mean scores of those who did not receive any training on patient safety.

**Table-III:** Positive response percentages of patient safety culture sub dimensions according to whether the nurses received patient safety training (n=316).

Dimensions	Trained (n=220)	Not Trained (n=96)	U-test
	Median (%25p.-%75p.)	Median (%25p.-%75p.)	p value
Overall perceptions of patient safety	0.75(0.50-1.00)	0.75(0.50-1.00)	0.414
Teamwork across hospital units	0.50(0.25-0.75)	0.50(0.25-0.75)	0.279
Hospital handoffs and transitions	0.75(0.50-0.93)	0.50(0.25-0.75)	0.017
Manager expectations and actions promoting safety	0.50(0.00-0.75)	0.25(0.06-0.50)	0.421
Organizational learning and continuous improvement	0.66(0.33-1.00)	0.66(0.33-1.00)	0.175
Teamwork within units	1.00(0.75-1.00)	1.00(0.75-1.00)	0.662
Communication openness	0.33(0.33-0.66)	0.33(0.33-0.66)	0.859
Feedback and communication about error	0.66(0.33-1.00)	0.33(0.33-0.66)	0.201
Non-punitive response to error	0.00(0.00-0.33)	0.33(0.00-0.33)	0.661
Staffing	0.25(0.25-0.50)	0.25(0.25-0.50)	0.289
Management support for patient safety	0.33(0.00-0.66)	0.33(0.00-0.66)	0.497
Frequency of events reported	0.00(0.00-0.66)	0.00(0.00-0.00)	0.003

## DISCUSSION

In this study, among the 12 dimensions of HSOPSC, the highest percentage of positive responses was obtained for “teamwork within units” and “nonpunitive response to error” had the lowest percentage. These results supported the previous studies on the subject^13^,^14^ (Table-II).

The results of this study revealed that 69.6% of the nurses stated that they received patient safety training as part of their in-service training programs. The percentage of the positive responses given by the nurses, who received in-service training for the dimensions “handoffs and transitions” and “frequency of events reported”, was found to be higher than percentage of those who did not receive any training on patient safety (Table-III). Çiftlik et al.,^15^ reported that success rate of the nurses on patient safety increased from 59% to 91.2% following a training offered to the medical staff. The findings of this study also indicated that in-service training improved awareness of nurses regarding patient safety.

About 43% of the nurses considered the patient safety in their units as “acceptable”. This result was similar to other studies conducted in Turkey.^16^,^17^ However, in a research conducted by the AHRQ,^14^ the nurses’ assessments about their units were determined to be “very good” in terms of patient safety.

Patient safety culture is crucial for prevention and correction of errors.^18^ The results of this study indicated that 88.0% of the nurses did not fill out a case report on patient safety in the last 12 months. In his study, El-Jardali et al.,^19^ determined that 57.2% of the nurses did not report any case on patient safety. Similarly, 81.5% of the nurses in Çakır and Tütüncü’s study were found not to have documented any incident on patient safety.^17^

The nurses were found to attribute the incidents of potential threat to patient safety due to the following reasons: heavy workload (55.8%), working in a stressful atmosphere (22.3%), and dealing with patients who are in need of specialized, complicated care (21.9%) (Table-I). In other studies conducted on patient safety, the nurses referred to unreadable handwriting, heavy workload, inadequate number of medical staff, long working hours, handling irrelevant tasks, inexperience, negligence, exhaustion, burnout, and stress as the causes of medical errors.^4^,^20^,^21^ The results obtained in this study had similarity with results of the previous study and suggested that the working conditions and heavy workload increased tendency of the nurses to make errors.

## CONCLUSION

In accordance with the above mentioned results, it is argued that through more frequent in-service trainings, awareness of the nurses regarding patient safety should be raised and their related knowledge should be kept up-to-date. In addition, number of studies on determination of patient safety culture should be increased and more comprehensive practice-based studies should be undertaken.
